# Effect of *ABCB1* and *ABCC3* Polymorphisms on Osteosarcoma Survival after Chemotherapy: A Pharmacogenetic Study

**DOI:** 10.1371/journal.pone.0026091

**Published:** 2011-10-07

**Authors:** Daniela Caronia, Ana Patiño-Garcia, Antonio Peréz-Martínez, Guillermo Pita, Leticia Tais Moreno, Marta Zalacain-Díez, Blanca Molina, Isabel Colmenero, Luis Sierrasesúmaga, Javier Benítez, Anna Gonzalez-Neira

**Affiliations:** 1 Human Genotyping Unit-CeGen, Spanish National Cancer Research Centre, Madrid, Spain; 2 Department of Pediatrics, University of Navarra and University Clinic, Pamplona, Spain; 3 Pediatric Oncology Department, Universitary Children's Hospital Niño Jesus, Madrid, Spain; 4 Human Genetics Group, Human Cancer Genetics Programme, Spanish National Cancer Research Centre, Madrid, Spain; National Cancer Center, Japan

## Abstract

**Background:**

Standard treatment for osteosarcoma patients consists of a combination of cisplatin, adriamycin, and methotrexate before surgical resection of the primary tumour, followed by postoperative chemotherapy including vincristine and cyclophosphamide. Unfortunately, many patients still relapse or suffer adverse events. We examined whether common germline polymorphisms in chemotherapeutic transporter and metabolic pathway genes of the drugs used in standard osteosarcoma treatment may predict treatment response.

**Methodology/Principal Findings:**

In this study we screened 102 osteosarcoma patients for 346 Single Nucleotide Polymorphisms (SNPs) and 2 Copy Number Variants (CNVs) in 24 genes involved in the metabolism or transport of cisplatin, adriamycin, methotrexate, vincristine, and cyclophosphamide. We studied the association of the genotypes with tumour response and overall survival. We found that four SNPs in two ATP-binding cassette genes were significantly associated with overall survival: rs4148416 in *ABCC3* (per-allele HR = 8.14, 95%CI = 2.73-20.2, *p-value* = 5.1×10^−5^), and three SNPs in *ABCB1*, rs4148737 (per-allele HR = 3.66, 95%CI = 1.85–6.11, *p-value* = 6.9×10^−5^), rs1128503 and rs10276036 (r^2^ = 1, per-allele HR = 0.24, 95%CI = 0.11–0.47 *p-value* = 7.9×10^−5^). Associations with these SNPs remained statistically significant after correction for multiple testing (all corrected *p-values* [permutation test] ≤0.03).

**Conclusions:**

Our findings suggest that these polymorphisms may affect osteosarcoma treatment efficacy. If these associations are independently validated, these variants could be used as genetic predictors of clinical outcome in the treatment of osteosarcoma, helping in the design of individualized therapy.

## Introduction

Osteosarcoma is the most frequent malignant bone tumour in children and adolescents. Standard treatment of osteosarcoma is based on a combination of different drugs: neoadjuvant therapy with methotrexate, cisplatin, and adriamycin followed by surgery and post-operative chemotherapy (methotrexate, cisplatin, adriamycin, cyclophosphamide, and vincristine). Despite this, approximately 30% of patients relapse or develop metastasis [1].

Clinical response to chemotherapeutics is a complex trait that is influenced by genetic and environmental factors. Anticancer therapies have a narrow therapeutic range so that a higher concentration in the patient's body causes toxicity and a lower concentration reduces the efficacy of the drug. Interindividual differences in pharmacokinetics and pharmacodynamics determine the global response and toxicity profile of each drug. In this process, the genes involved are the ones that control drug absorption, distribution, metabolism, and excretion. The majority of metabolism reactions are catalyzed by the cytochrome P450 (CYP) enzymes [2]. Many chemotherapeutic agents are also metabolized by glutathione S-transferases (GSTs), phase II detoxification enzymes that catalyse the conjugation of glutathione (GSH) to a wide variety of xenobiotics [3]. Two types of transport superfamilies, ATP-binding cassette (ABC proteins) [4] efflux pumps and solute-linked carrier (SLC) influx proteins [5] are responsible for the majority of drug transport [6]. Most of the drug metabolizers and transporters contain many genetic polymorphisms, which might cause large interindividual variability in the plasma concentration of drugs.

Pharmacogenetic studies have shown that germline polymorphisms in genes related to drug metabolism and transport can have a major effect on the pharmacokinetics and pharmacodynamics of these drugs [6]. We previously performed a study of the nucleotide excision DNA repair pathway in relation to response to cisplatin and observed an association between osteosarcoma outcome and a polymorphism in *ERCC2* gene [7]. Nevertheless, a large portion of the interindividual variability in therapeutic response remains unexplained.

Since mechanisms of transport and metabolism are shared between drugs, genetic variation could affect the bioavailability of more than one drug when they are used in combination, thus affecting the global response to treatment or causing adverse drug events. To address this, pharmacogenetic studies will need to focus on integration of multiple drug pathways to allow a more complete analysis of genetic factors influencing drug efficacy and toxicity.

In the current study, we studied a comprehensive set of SNPs and CNVs that characterize the genetic variation of the multiple metabolic and transport pathways of drugs used in osteosarcoma treatment and their association with drug response.

## Methods

### Patients, treatments, and clinical variables

One hundred and two consecutive patients diagnosed with osteosarcoma at the University Clinic of Navarra, Pamplona, Spain, between 1986 and 2009 were enrolled in this study. All samples were obtained with written informed consent from patients, their parents, or both. Ethical approval of the study was granted by the Ethics Committee of the University Clinic.

Patients were treated preoperatively with intravenous (i.v.) adriamycin (3 courses at 25–30 mg/m^2^/day for 3 days), i.v. methotrexate (4 courses of up to 14 g/m^2^/day for 1 day) and intra-arterial cisplatin (3 courses at 35 mg/m^2^/day for 3 days). After surgery, the adjuvant chemotherapy included methotrexate (10 g/m^2^/day for 1 day and folinic acid rescue) and alternate cycles of i.v. cisplatin/adriamycin or i.v. actinomycin D (0.45 mg/m^2^/day for 3 days), cyclophosphamide (500 mg/m^2^/day for 3 days), and vincristine (1.5 mg/m^2^/day for 1 day) for up to 48 weeks of treatment.

Response to treatment was determined histologically as the percentage of necrosis induced in the tumour after neoadjuvant chemotherapy. Patients with less than 90% necrosis were classified as poor responders and those with 90% necrosis or more, as good responders [8]. Overall survival was considered as the time from tumour diagnosis to death. Event-free-survival (EFS) was considered as the time from tumour diagnosis to the first of disease recurrence, development of lung or bone metastases, and/or death. Patients who were alive at the last follow-up evaluation (January, 2010) were censored at that time. Other clinical data including age, sex, tumour location, metastatic events (both at diagnosis and during follow-up) and relapses (disease recurrence in the same bone) were systematically recorded from the clinical records. Only conventional high-grade osteosarcomas were included, regardless of metastatic stage at diagnosis.

### DNA extraction and quantification

Genomic DNA was extracted from peripheral blood lymphocytes using standard phenol-chloroform extraction protocols. DNA was quantified using PicoGreen (Invitrogen Corp., Carlsbad, CA).

### Candidate genes and selection of polymorphisms

A total of 24 candidate genes reported to be involved in the metabolism or influx/efflux of the five drugs (cisplatin, adriamycin, methotrexate, vincristine, and cyclophosphamide) were selected, based on the information available in the Pharmacogenomics Knowledge database PharmaGKB (www.pharmgkb.com). These genes encode the following proteins:

*Transporters*: ABCA3, ABCB1, ABCC1, ABCC2, ABCC3, ABCC4, ABCG2, ABCC6, SLC31A1, SLCO6A1, SLC19A1;

*Phase I metabolism enzymes*: MPO, SOD1, ALDH1A1, CYP3A4, 3A5, 2A6, 2B6, 2C8, 2C19, 2C9;

*Phase II metabolism enzymes:* GSTM1, GSTP1 and GSTT1.

SNPs were selected across all these genes except for the *GSTT1* gene. CNVs were studied in the *GSTT1* and *GSTM1* genes.

TagSNPs for the selected genes were defined using Haploview software v.4.0 (http://www.broad.mit.edu/mpg/haploview) with an r^2^ threshold of 0.8 and a minimum minor allele frequency of 0.05. All defined tagSNPs were selected for possible genotyping except those in the *ABCC4* gene, for which an excessively elevated number of tagSNPs was defined and so a subset of 57 of these were selected.

In addition, both SNPs with potentially functional effects (causing amino acid changes, potentially causing alternative splicing, in the promoter region, in putative transcription factor binding sites, or disrupting miRNAs and their targets) identified using the bioinformatics tool PupaSuite (http://bioinfo.cipf.es/pupasuite/www/index.jsp), and other functional SNPs already described in the literature were selected.

This preliminary list of SNPs was filtered using as criteria suitability for the Illumina genotyping platform (selecting only those with an assay score >0.6, associated with a high success rate) and minor allele frequencies (MAFs) of at least 5%.

A final number of 366 SNPs relevant to this study was included in an oligonucleotide pool assay for analysis using the Illumina Veracode technology (Illumina Inc., San Diego, CA).

### SNP Genotyping

300 ng of DNA for each sample were genotyped using the GoldenGate Genotyping Assay with Veracode technology according to the published Illumina protocol. Data were analysed with GenomeStudio software for genotype clustering and calling. We excluded from further analysis SNPs with a call rate <0.95 and SNPs that deviated from Hardy-Weinberg equilibrium.

Duplicate samples and CEPH trios (Coriell Cell Repository, Camden, NJ) were genotyped across the plates. SNPs showing Mendelian allele-transmission errors or showing discordant genotypes were excluded from the analysis.

### *GSTM1* and *GSTT1* copy number assays

*GSTT1* and *GSTM1* copy number was calculated using Taqman Copy Number Assays (Hs00010004_cn probe for *GSTT1* and Hs02575461_cn for *GSTM1*, Applied Biosystems, Foster City, CA) following the manufacturer's protocol on an ABI PRISM 7900 Sequence Detection System (Applied Biosystems). *RNAse P* was used as the reference in TaqMan Copy Number Reference Assays. Data were analyzed using absolute quantification of resulting Ct values generated on the sequence detection system. Copy number was estimated using the CopyCaller 1.0 software (Applied Biosystems). Each sample was evaluated in triplicate.

### Statistical analysis

Associations between tumour response and genotypes was assessed using logistic regression analysis [9], comparing genotype frequencies in poor responders versus good responders and estimating odds ratios (OR). Homozygotes for the most frequent allele were used as the reference group. SNPs were also assessed in relation to overall survival and event free survival (EFS) using Cox regression analysis [10]. Metastasis at diagnosis (no, yes) was included as covariate in multivariable logistic regression and Cox regression analyses.

A permutation test was used to estimate *p-values* corrected for multiple testing. Each replicate consisted of randomly assigning the set of three variables, response to treatment, survival status and analysis time, across subjects and then carrying out the association analyses for each of the 348 successfully genotyped variants and each of the two outcomes (response to treatment and survival). The minimum *p-value* of these 696 ( = 348×2) tests was then recorded. Ten thousand such replicates were carried out and the corrected *p-values* were estimated as the proportion of replicate *p-values* less than the corresponding unadjusted *p-value*. Reported *p-values* are uncorrected for multiple testing, unless otherwise stated. Only SNP associations with corrected *p-values* <0.05 were considered statistically significant.

Haplotypes containing rs1045642, rs2032582 and rs1128503 in *ABCB1* were inferred by PHASE software, version 2.0. Association with overall survival was then assessed using Cox-regression analysis. Kaplan-Meier curves were generated using SPSS (version 15.0, SPSS Inc., Chicago, IL, USA). All other analyses were carried out using PLINK or R (version 2.6.0.2).

## Results

### Patients

The main clinical data for the 102 osteosarcoma patients are presented in Table 1. The median age at diagnosis was 14 years (range 3 to 34 years). At the time of diagnosis, 21% of the patients already presented metastasis, while 22% developed metastasis during follow-up. The median follow-up time was 231 months (range 3–303).

**Table 1:** Clinical characteristics of osteosarcoma patients (*N* = 102).

	Patients	
	*N*	%
Age at diagnosis (years)		
Median	14.8	
Range	3–34	
Sex		
Female	45	44.1
Male	57	55.9
Location		
Femur	51	50.0
Tibia	38	37.3
Arm	7	6.9
Central	6	5.9
Response to treatment		
Good	52	57.1
Poor	39	42.9
Metastasis		
No	59	57.8
At diagnosis	21	20.6
At follow up	22	21.6
Status		
Alive	72	71.3
Dead	29	28.7
Relapse		
No	85	83.3
Yes	17	16.7

Response to treatment (necrosis) data were available for 91 patients and overall survival data were available for 101 patients. The percentage of good responders to therapy was 54 and the median overall survival was 219 months.

Of the clinical variables analyzed, metastasis at diagnosis was found to be associated with increased risk of death (HR = 2.92, 95%CI = 1.35–6.28, *p-value* = 0.006).

### Association between polymorphisms and clinical data

A total of 346 SNPs out of 366 and two CNVs were successfully analyzed. Eleven patients were removed for low genotyping call rate (<95%), so finally 91 patients were successfully analyzed.

**Figure 1:**
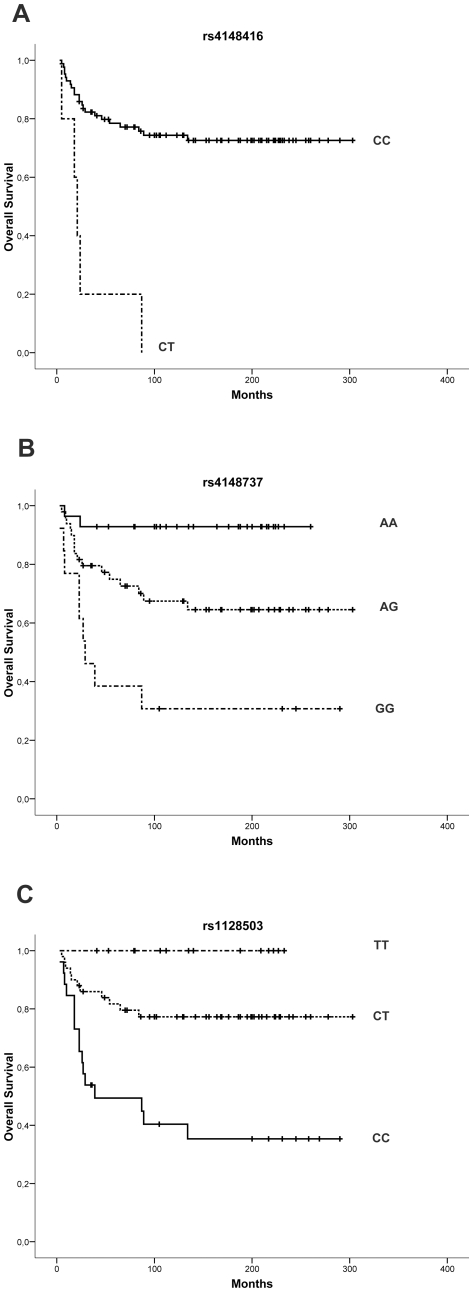
Kaplan-Meier survival curves for osteosarcoma patients according to genotype for (A) rs4148416 in *ABCC3* (X^2^ = 21.4, *p-value* = 3.8×10^−6^); (B) rs4148737 in *ABCB1* (X^2^ = 18.4, *p-value* = 1.0×10^−4^); and (C) rs1128503 or rs10276036 in *ABCB1* (X^2^ = 20.9, *p-value* = 2.9×10^−5^ *).*

Results from the analysis of overall survival are presented in Table 1 and Figure 1. We identified four SNPs (two of them in complete linkage disequilibrium) in two genes that were associated with overall survival. The T allele of the synonymous SNP rs4148416 (G1013G) in the *ABCC3* gene was associated with higher risk of death (per-allele HR = 8.14, 95%CI = 2.73–20.2, *p-value* = 5.1×10^−5^, Figure 1A). In particular, the estimated five-year survival rate for patients carrying the CC genotype of rs4148416 was 78% compared to 20% for heterozygous patients.

The G allele of s4148737, an intronic SNP located in the *ABCB1* gene, was associated with poorer overall survival (per-allele HR = 3.66, 95%CI = 1.85–6.11, *p-value* = 6.9×10^−5^,) Figure 1B). The estimated five-year survival rate for patients carrying the common AA genotype was 93% compared to 38% for patients homozygous for the G allele. The minor alleles of two other SNPs (rs1128503 and rs10276036) in this gene, in complete linkage disequilibrium (LD, r^2^ = 1.0), and in partial LD with rs4148737 (r^2^ = 0.48), were also associated with better overall survival (per-allele HR = 0.24, 95%CI = 0.11–0.47, *p-value* = 7.9×10^−5^, Figure 1C). For these two SNPs, the estimated five-year survival rate for common homozygotes was 49% compared to 100% for patients homozygous for the rare allele. These results did not change substantially after adjusting for metastasis at diagnosis (Table 2). All three associations remained statistically significant after correction for multiple testing (corrected *p-value*≤0.030).

**Table 2:** Genes and polymorphisms associated with overall survival (OS) and event free survival (EFS).

SNP	Genotype	N	5-year	OS HR*	OS Adjusted**HR	EFS HR
			survival rate	(95%CI)	(95%CI)	(95%CI)
				*P-value*	*P-value*	*P-value*
***ABCC3***						
*rs4148416*	CC	85	78%			
	CT	5	20%			
	per allele T			8.14	7.25	6.33
				(2.73–20.2)	(2.62–20.1)	(1.79–12.7)
				0.000051	0.00014	0.00028
***ABCB1***						
*rs4148737*	AA	28	93%			
	AG	49	75%			
	GG	13	38%			
	per allele G			3.66	2.83	2.60
				(1.85–6.11)	(1.56–5.12)	(1.24–3.22)
				0.000069	0.00061	0.00051
*rs1128503*	CC	26	49%			
	CT	50	82%			
	TT	14	100%			
	per allele T			0.24	0.27	0.42
				(0.11–0.47)	(0.13–0.54)	(0.29–0.81)
				0.000079	0.00023	0.0021
*rs10276036*	TT	26	49%			
	TC	50	82%			
	CC	14	100%			
	per allele C			0.24	0.27	0.42
				(0.11–0.47)	(0.13–0.54)	(0.29–0.81)
				0.000079	0.00023	0.0021

*HR: Hazard Ratio.

**Analysis adjusted for metastasis at diagnosis.

We also studied these SNPs in relation to event-free survival (EFS) and highly consistent results were observed (Table 2). None of the SNPs analyzed were significantly associated with tumour response after correction for multiple testing.

A combination of three SNPs located in *ABCB1* (rs1045642 / rs2032582/ rs1128503) was previously described as putatively functional in several studies [4],[11],[12]. We explored whether there is a haplotype formed by these three SNPs that is more significantly associated with survival in these patients. We observed two frequent haplotypes, one formed by the combination of the three common alleles (CGC) with a frequency of 0.47 and the other comprising the three rare alleles (TTT) with a frequency of 0.39. Considering CGC as reference, the TTT haplotype was associated with better survival (HR = 0.31, 95%CI = 0.15–0.62, *p-value* = 0.001). The other haplotypes observed had lower frequency and were not statistically significantly associated with survival. Nevertheless, the estimated HR for the two haplotypes containing the rare T allele in rs1128503 (CGT and CTT) were consistent with it having a protective effect (HR = 0.38 and 9.78×10^−6^, respectively) in contrast with the other two haplotypes containing the C wild-type allele (TGC and TTC; HR = 1.26 and 1.95, respectively). These results suggested that the haplotypes were no more informative than the rs1128503 SNP alone.

Regarding the CNVs analyzed, genotype data for *GSTM1* and *GSTT1* were available for 98 and 99 patients, respectively. The frequency of the homozygous gene deletion was 52% (51 patients) for *GSTM1* and 19% (19 patients) for *GSTT1*. There was no evidence that either of these two polymorphisms were associated with any of the clinical outcomes considered.

## Discussion

This study assessed 346 SNPs and 2 CNVs in 24 key genes involved in platinum, adriamycin, methotrexate, vincristine, and cyclophosphamide pathways, and is the most comprehensive pharmacogenetic study in osteosarcoma patients to date.

The use of large scale genotyping methods to screen multiple drug pathways has allowed us to identify four SNPs (two of them in total LD) that are highly associated with overall survival and that might be useful prognostic markers in these patients.

To our knowledge, the majority of the pharmacogenetic studies are biased towards candidate polymorphisms in pharmacogenetic genes already described as functional in previously published data. However, given the complexity and the lack of understanding of both genetic variation effects and regulation of chemotherapy action, these polymorphisms explain only a portion of the observed phenotypic variability in the drug outcome. In our study, we have assessed all genes well-known to be involved in the metabolism and transport pathways of the drugs used in osteosarcoma treatment. In order to avoid any bias, we selected not only already described and potentially functional polymorphisms, but also tagSNPs, to obtain a more comprehensive view and to detect novel markers that could play a role in the interindividual differences observed of outcome risk in osteosarcoma patients.

*ABCC3* is a member of the multidrug resistance protein (MRP) family and is expressed in liver, gallbladder, kidney, and gut [13],[14]. Main substrates of *ABCC3* are bile salts [15], but it also transports anticancer drugs, among which methotrexate [16], [17]. Vincristine, doxorubicin, and cisplatin have also been suggested to be *ABCC3* substrates, but there are no clear results [17], [18]. The expression of *ABCC3* mRNA has been related to drug resistance [16] but to date only limited studies have been published studying polymorphisms in *ABCC3*. Only promoter SNPs and non-synonymous SNPs have been investigated as potentially functional variants [19], [20], but, to our knowledge, there are no studies showing association between these genetic *ABCC3* variants and survival after treatment in cancer patients. In the present study we found that SNP rs4148416 in this gene was associated with an 8-fold risk of low survival and this is the first evidence of its clinical relevance. This SNP leads to a synonymous change (G1013G) at exon 22. The other SNPs we found associated with osteosarcoma survival are located in the *ABCB1* gene. This gene is well-known and encodes a P-glycoprotein, an ATP-driven efflux pump, that is overexpressed in many tumours and confers multidrug resistance [21]. Of the drugs administered to these patients, both doxorubicin and vincristine are transported by this pump [22]. There are three variants in LD that have been studied in detail: 2677G>T/A (rs2032582), 3435C>T (rs1045642) and 1236C>T (rs1128503). The first is a non-synonymous change, while the other two are synonymous. These three SNPs have been studied both individually and as a haplotype, but the results have been inconsistent [4] [23] [11] [24]. The rare allele of rs1045642 has been reported to be associated with reduced P-glycoprotein activity, both alone and in combination with the rare alleles of rs2032582 and rs1128503 [23], [25]. These latter two variants have also been reported to have independent functional effects in different studies [12], [25], [26], [27]. However, it is still not clear which is/are the functional variant/s in this gene.

In our study, of these three variants, only 1236T>C (rs1128503) was strongly associated with survival in our series of patients. This SNP leads to a synonymous change at residue 412 of the protein and is well-known but there is no clear consensus on its functional significance [28]. Some studies found increased response in the presence of the T allele [29], [30] while others found the opposite [31], or no genetic effect [32]. In our study, the T allele was associated with increased survival. To see if the association found was due to this single variant or to a combination of the three already described polymorphisms we also analyzed the effect of the haplotypes on overall survival. The results were consistent with a single main effect for rs1128503. Consistent results were reported by Balcerczak and colleagues in colorectal cancer patients [12].

We found another SNP, rs10276036, associated with survival that was in complete LD with the previous one. It is located in intron 9 and has been linked with reduced area under the curve (AUC) of SN-38, the active metabolite of CPT-11, [33]; however its functional significance is unknown. The effect observed could be explained by its correlation with rs1128503, rather than by rs10276036 itself. Apart from the already described SNPs, we found a polymorphism located in intron 17 (rs4148737) also strongly associated with survival. This SNP was in low LD with the other two SNPs. Further functional studies are needed to elucidate which is the causal variant in the *ABCB1* gene.

We hypothesize that the variants associated with overall survival could have an effect on the efflux of the drugs used in the treatment of osteosarcoma, thus impairing the response to the treatment and therefore the overall survival. Although we did not find a statistical association between these variants and tumour response, this could be explained by the fact that tumour response is evaluated after neoadjuvant therapy and at this point patients have been treated exclusively with methotrexate, cisplatin, and adriamycin. Therefore, the tumour necrosis data does not evaluate the effect of the vincristine and cyclophosphamide drugs used after surgery. Since ABCB1 is known to transport vincristine, ABCC3 could also be involved in this process [18], [34], and the transport mechanisms for cyclophosphamide are still unknown, we postulate that genetic variation in these transporters could play a role in the effectiveness of the whole treatment and influence the overall survival.

In conclusion, this study identified four significant SNPs in two drug transporter genes associated with overall survival in osteosarcoma patients. After validation in large and well-defined sets of patients to confirm the associations, these variants could be useful as prognostic markers in these patients.

Furthermore, the approach used in this study, integrating multiple drug pathways and studying an increased number of polymorphisms, may be extended to future pharmacogenetic studies to provide a wide scenario of genetic factors influencing drug efficacy and toxicity. The applicability of high-throughput gene chips that allow simultaneous analysis of multiple polymorphisms will facilitate research in this field.
